# Contrasting Plasticity in Ovariole Number Induced by A Dietary Effect of the Host Plants between Cactophilic *Drosophila* Species

**DOI:** 10.3390/insects7020021

**Published:** 2016-05-21

**Authors:** Daniela Peluso, Eduardo M. Soto, Lucas Kreiman, Esteban Hasson, Julián Mensch

**Affiliations:** IEGEBA-CONICET-UBA, DEGE, Facultad de Ciencias Exactas y Naturales, Universidad de Buenos Aires, Buenos Aires 1428, Argentina; dany.peluso@hotmail.com (D.P.); edusoto@ege.fcen.uba.ar (E.M.S.); lucaskreiman@yahoo.com.ar (L.K.); ehasson@ege.fcen.uba.ar (E.H.)

**Keywords:** phytophagous insects, developmental plasticity, female reproductive capacity

## Abstract

Under the preference-performance hypothesis, natural selection will favor females that choose oviposition sites that optimize the fitness of their offspring. Such a preference-performance relationship may entail important consequences mainly on fitness-related traits. We used the well-characterized cactus-*Drosophila* system to investigate the reproductive capacity in the pair of sibling species *D. buzzatii* and *D. koepferae* reared in two alternative host plants. According to our hypothesis, ovariole number (as a proxy of reproductive capacity) depends on host plant selection. Our results indicate that the capacity of *D. buzzatii* showed to be mild, only increasing the number of ovarioles by as much as 10% when reared in its preferred host. In contrast, *D. koepferae* exhibited a similar reproductive capacity across host cacti, even though it showed a preference for its primary host cactus. Our study also revealed that *D. buzzatii* has a larger genetic variation for phenotypic plasticity than its sibling, although ovariole number did not show clear-cut differences between species. We will discuss the weak preference-performance pattern observed in these cactophilic species in the light of nutritional and toxicological differences found between the natural host plants.

## 1. Introduction

**Figure 1:**
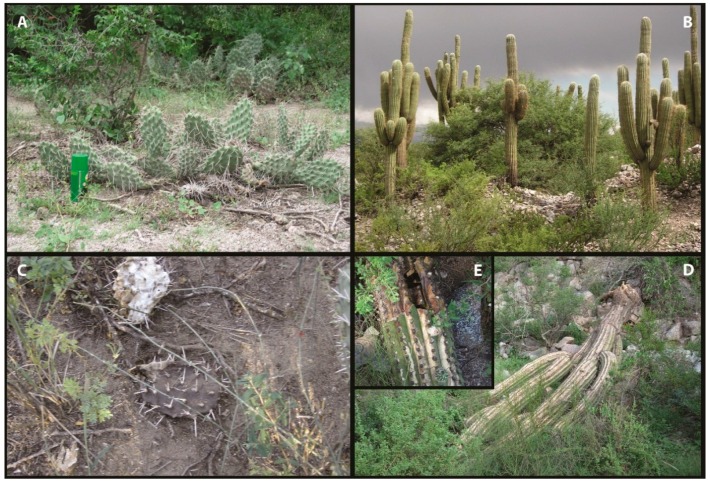
Natural host plants of cactophilic *Drosophila* in Argentina. (**A**) *Opuntia sulphurea*; (**B**) *Trichocereus terscheckii*; (**C**) Rotting cladodes of *O. sulphurea*; (**D**) Decaying tissues of *T*. *terscheckii*; (**E**) Zoom in rotting tissues of *T*. *terscheckii*.

Ovariole number variation is one of the most important indicators of the reproductive effort made by females in relation to resource availability for offspring [1,2]. A striking variation in the number of these egg-producing structures is found among species and populations as a consequence of distinct evolutionary histories [2,3,4,5]. Moreover, the number of ovarioles exhibits strong phenotypic plasticity in response to changing environmental conditions during juvenile development [6,7]. Specifically, larval nutrition impacts ovariole morphogenesis in a way that it can affect the amount of developing units. Flies reared on food with less yeast are smaller and have fewer ovarioles than those reared with more yeast [7,8,9]. It has been recently suggested that nutritional plasticity of ovariole number has an adaptive value [10]. Only generalist *Drosophila* species, such as *D. melanogaster* and *D. simulans*, changed the number of ovarioles under diet variation. In contrast, specialist species, such as *D. sechellia* and *D. erecta*, exhibit similar ovariole number across different diets. Such adaptive plastic responses allow generalist species to express advantageous phenotypes in a broader range of environments, unlike specialist species, which show a lack of environmental sensitivity. The *Drosophila repleta* species group is a monophyletic group of Neotropical flies that has diversified in the Western Hemisphere, by adopting a cactophilic lifestyle that allows them to thrive in the American deserts [11,12,13]. Females lay eggs in necrotic cactus tissues (Figure 1), which is the particular environment in which larvae feed until pupation. This diet not only consists of carbohydrates and lipids derived from decaying cactus tissues [14,15], but also proteins of yeasts and bacteria involved in the decaying process [16]. Once the flies emerge, adults feed on decaying cacti. This well-characterized cactus-yeast-*Drosophila* system is an excellent model to study the impact of natural hosts on the reproductive capacity of flies [17,18].

The South American *Drosophila buzzatii* and *Drosophila koepferae* are two sibling cactophilic species that use alternative types of cacti as main natural hosts: Prickly pears of the genus *Opuntia* and columnar cacti of the genera *Cereus* and *Trichocereus* (Figure 1). Previous work has shown that host plants are species-specific toxicological [19,20] and nutritional [14,15] challenges to developing larvae. Even though there is some niche overlap, *D. buzzatii* is mainly associated to *Opuntia sulphurea*, whereas *D. koepferae* uses *Trichocereus terscheckii* as its main natural resource in most of both these species’ distribution ranges [21]. These contrasting preferences result in plastic performances in alternative breeding resources in terms of morphological [22,23,24], behavioral [17,18] and life-history traits [18,20,25]. Importantly, over the past few hundred years, *D. buzzatii* has colonized the Canary Islands, several countries of the Mediterranean basin, Ethiopia, and Australia [26,27]. Such sub-cosmopolitan distribution is the result of its association with prickly pears of the genus *Opuntia*, which themselves spread by human intervention and became established [27]. It has been recently reported, however, the first record of *D. buzzatii* emerging from a non-cactus host in western Argentina [28]. Moreover, in recent field collections, we found that *D. buzzatii* can emerge from rotting grapes (*Vitis vinifera*), asserting the capacity of this species to exploit other nutritional resources than cacti (E.M.S., E.H. and J.M., unpublished results [29]). Thus, the ecological niches between these cactophilic *Drosophila* differ depending on oviposition substrate choice, the abundance of substrates, and the nutritional content for larval growth.

In this work, we will study if oviposition host-preference correlates with a phenotypic plasticity on the number of ovarioles by a dietary effect of the host. Under the preference-performance hypothesis, natural selection will favor females that lay eggs on sites that optimize the fitness of their offspring [30,31,32]. Given that females of *D. buzzatii* prefer to lay their eggs on *O. sulphurea*, either in lab or in field experiments [18,33], we expect to have an increased ovariole number in the females raised in this cactus, as compared to flies reared in columnar *T. terscheckii*. In contrast, since females of *D. koepferae* have a mild preference between these two natural resources [18], we predict a similar ovariole number as a consequence of breeding in alternative cacti. Moreover, since *D. buzzatii* has a wider ecological niche than *D. koepferae,* we expect a greater genetic variation for phenotypic plasticity in ovariole number in the former species.

## 2. Experimental Section

### 2.1. Fly Collections and Stock Maintenance

Fly collections were carried out in San Agustín del Valle Fértil Natural Reserve (30°3′ S, 67°3′ W, San Juan Province, Argentina) in March 2014, Ruinas de Quilmes (26°3′ S, 66°0′ W, Tucumán Province, Argentina) and Vipos (26°4′ S, 65°4′ W, Tucumán Province, Argentina) in March 2011. Two cactus species, the prickly pear *O. sulphurea* and the columnar *T. terscheckii* are the most abundant hosts in the areas (Figure 1). Flies were collected by net sweeping on fermented banana baits, sexed upon arrival to the laboratory and used to set isofemale lines (lines from here on) by placing individual females in vials containing 5 mL of Instant *Drosophila* Medium (Carolina Biological Supplies, Burlington, NC, USA). Lines were identified to species by examining the genitalia (*aedeagus*) of several progeny males of each line, since females of these species are morphologically indistinguishable [34]. To determine within-species variation, we evaluated 5 isofemale lines (lines from hereafter) from each species. Eight lines (four of each species) derived from the locality of Valle Fértil, and the fifth *D. buzzatii* and *D. koepferae* lines derived from Ruinas de Quilmes and Vipos, respectively.

### 2.2. Oviposition Preferences of D. buzzatii and D. koepferae Isofemale Lines

In order to check if the isofemale lines employed in the present study conserve their natural oviposition host-preference behavior, we carried out some experiments forcing flies to choose between *O. sulphurea* or *T. terscheckii*. We measured the number of eggs laid on each of the cactus within 24 h. See [18] for details.

### 2.3. Effect of Semi-Natural Diets on Ovariole Number

#### 2.3.1. Semi-Natural Diets

We also collected fresh materials of *O. sulphurea* and *T. terscheckii* in the same sampling locality (Valle Fértil, San Juan, Argentina) to be used in the preparation of two types of “semi-natural” media. Once back in the lab, the fresh materials were stored at −20 °C. For the preparation of the two cactus media, pieces of each species defrosted cactus were weighed and ground in a blender and 10 mL of the liquefied cactus plus 1 g of dry yeast per 1 g of cactus were poured into standard *Drosophila* vials. The vials were autoclaved and allowed to cool down before experiments.

#### 2.3.2. Ovariole Number

**Figure 2:**
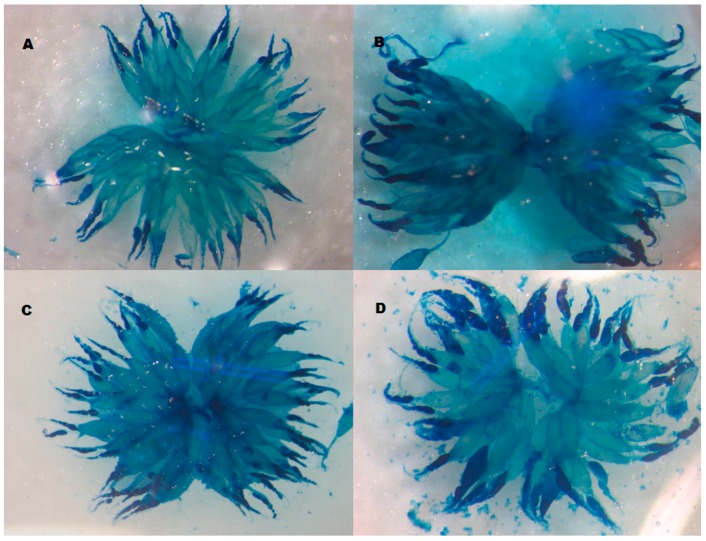
Ovaries stained with methylene blue. (**A**,**B**) *D. buzzatii* females reared in *O. sulphurea* and *T. terscheckii*, respectively and (**C**,**D**) *D. koepferae* females reared in *O. sulphurea* and *T. terscheckii,* respectively.

For each isofemale line, 300 pairs of sexually mature flies were placed in oviposition chambers for 8 h. Eggs were allowed to hatch and batches of 40 first-instar larvae were transferred to culture vials containing alternative semi-natural media in order to control larval density in the vials. The vials were placed in an incubator at 25 ± 0.5 °C, under a 12:12 h light:dark photoperiod and 60%–70% of humidity. Groups of 10 females were held with an equal number of males in vials at 25 °C for 7 days. Then, females were stored at −80 °C until dissection and ovariole number determination. Prior to ovary dissections, we measured wing length (see below), a common measure of body size, in order to account for size variation between species and rearing conditions. For ovariole number estimation, each female was placed in a drop of phosphate-buffered saline (PBS) buffer containing 1% of methylene blue (Sigma, Buenos Aires, Argentina). The tip of the abdomen was pulled away in a posterior direction while the rest of the body was held using a second pair of forceps. Ovarioles were gently separated from each other using tungsten needles. Since methylene blue differentially dyes germarium and mature oocytes, we determined the number of ovarioles by counting germaria in both ovaries of each female (Figure 2). This method improves ovariole number estimation, since each ovariole holds a single germarium. We then counted the number of ovarioles in two replicates per combination of genotype by rearing condition (resulting in an average of 10 females for each combination of genotype by rearing cacti).

#### 2.3.3. Wing Length

Wing length is considered to be a proxy of body size in insects, in general [35] and in cactophilic *Drosophila*, in particular [23,36]. For this reason, prior to ovariole count, both wings of each female were removed and mounted on microscope slides. Right wing images were captured using a binocular microscope and an attached digital camera connected to a computer. For each wing, we scored wing length as the length of the second wing vein (see [23] for details), using the software TPSDIG v1.4 [37] to digitalize landmarks.

### 2.4. Plasticity Index

In order to compare the level of phenotypic plasticity between species and traits (body size and ovariole number) a Relative Distances Plasticity Index (RDPI) was estimated according to Valladares *et al.* [38]. This index gauges the absolute phenotypic distance between individuals of the same genotype grown in different cacti, divided by the sum of the two phenotypic values, which results in a number ranging from 0 (no plasticity) and 1 (high plasticity). A RDPI can be obtained for each species and traits as:

RDPI = ∑ (dij → i’j’/(xij + xi’j’))/N
(1)

where dij → i′j′ is the absolute phenotypic distance between individuals of the same genotype grown in different cacti, xij + xi′j′ is the sum of the two phenotypic values and N is the total number of phenotypic distances.

### 2.5. Effect of A Standard Diet (with No Cactus Additive) on Ovariole Number

Isofemale lines were reared in the lab with a standard diet (Instant *Drosophila* Medium (Carolina Biological Supplies)) for several generations until the beginning of this study. In order to evaluate carry-over effects of lab rearing on ovariole number, we performed two kinds of analyses: (1) comparing the number of ovarioles in flies fed a standard diet with flies fed each of the semi-natural diets; and (2) comparing the number of ovarioles in flies emerging from rotting cactus with flies fed each of the semi-natural diets (see the following section). Conditions of temperature, photoperiod and larval density were similar to the previous assays.

### 2.6. Effect of Natural Substrates on Ovariole Number

A second fly collection trip was carried out in the San Agustín del Valle Fértil Natural Reserve (30°3′ S, 67°3′ W, San Juan Province, Argentina) in March 2016. For this opportunity, we collected rotting stems of *T. terscheckii* and rotting cladodes of *O. sulphurea.* Substrates were wrapped in paper in the field and brought to the laboratory where they were placed in plastic containers. All the flies that emerged within the following two weeks after collection were aspirated daily and classified by species (see below). All the flies that emerged from the same container were kept in groups of 20 individuals per bottle for six days. This procedure ensured that the flies of both species reached sexual maturity and that all the females were inseminated. Subsequently, the flies were sorted by sex, and the females were used to establish isofemale lines. Several males of the progeny of each isofemale line were classified by species through the inspection of their genitalia. Finally, we counted the ovarioles of all the females that could be identified as belonging to *T. terscheckii* and *O. sulphurea* species. Overall, using this protocol, we were able to compare ovariole numbers of *D. buzzatii* and *D. koepferae* emerged from natural substrates with females reared on alternative semi-natural media.

### 2.7. Statistical Analysis

Ovariole number variation was analyzed by means of an analysis of co-variance (ANCOVA) using wing length as covariate with the model:

Y = μ + S + C + S × C + L (S) + L (S) × C + R (L × C) + WL + E
(2)

where μ is the overall mean, with Species (S) and Cactus (C) as a fixed effects (both with two levels), lines (L) nested in Species, Replicates (R) nested in Line by Cactus interaction, the Species by Cactus interaction (S × C), Wing Length (WL) as the covariate and the error (E) term. *Drosophila* species and Cactus are fixed factors, while Lines and replicates are random factors. Replicate factor was excluded from the model, since it did not increase the model goodness of fit. According to the experimental design, a significant cactus effect may be interpreted as phenotypic plasticity, while significant differences among isofemales lines (L) may be due to genetic differences. Additionally, a significant L × C interaction may be interpreted as a genotype-by-diet interaction indicating genetic variation for phenotypic plasticity.

## 3. Results

Before assessing the effect of semi-natural diets on ovariole number, we evaluated whether isofemale lines used in this study retained their natural oviposition host-preference behavior after generations of lab rearing. Our oviposition preference assay revealed significant differences between species in the proportion of eggs laid in each cactus (F_1,21_ = 22.16, *p* = 0.012) confirming that females of *D. buzzatii* and *D. koeperae* prefer to lay eggs on *O. sulphurea* (67%) and *T. terscheckii* (71%), respectively.

### 3.1. Effect of Semi-Natural Diets on Ovariole Number

**Figure 3:**
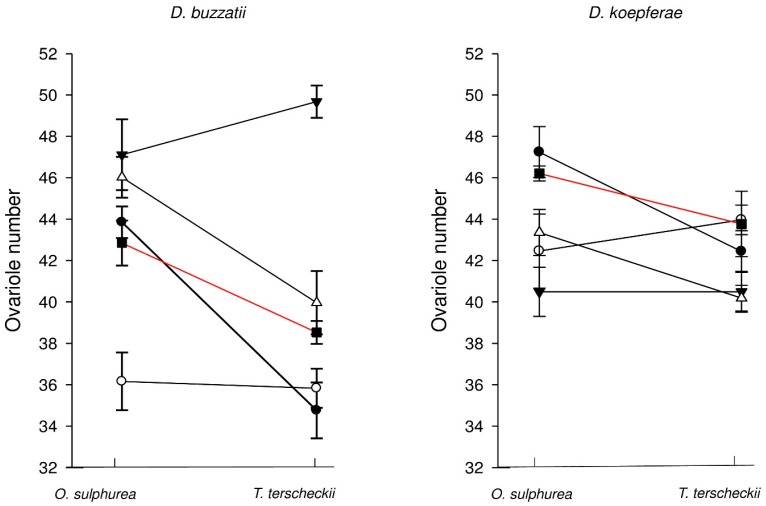
Norms of reaction of all genotypes reared in alternative cacti. Black reaction norms correspond to genotypes derived from the locality of Valle Fértil while red reaction norms correspond to genotypes derived from populations of different geographic origins.

The analysis of co-variance revealed a significant Line-by-Cactus interaction (Table 1), indicating that variation among lines in ovariole number depended on the rearing cactus. In other words, ovariole number scores of the lines tested varied across rearing cacti. Under our experimental design, these results can be interpreted as genetic variation for phenotypic plasticity on ovariole number. Importantly, wing length (as a proxy of body size) did not covariate with variation on ovariole number. We decided to perform an analysis of variance for each *Drosophila* species, since genotypes (Line effect) are nested in *Drosophila* species factor (Table 2 and Table 3). Only *D. buzzatii* females showed a significant genotype-by-cactus interaction, reflected in a 10% increment in mean ovariole number when reared in the preferred host, *O. sulphurea*, relative to females reared in *T. terscheckii* (Figure 3). In contrast, *D. koepferae* females exhibited similar ovariole number across rearing cacti (Figure 3). To investigate to what extent these patterns are due to the particular populations initially sampled, we studied ovariole number in two lines (one line of each species) derived from other geographic origin than the lines included in the analysis referred to above. Interestingly, these new genotypes showed comparable patterns of variation to the original populations (Figure 3), suggesting species’ differences in phenotypic plasticity for ovariole number.

Additionally, we used a relative distance plasticity index (RDPI) [38] to compare the degree of phenotypic plasticity between species and traits (body size and ovariole number). Values of RDPI ranged from 0.014 to 0.093. The analysis of RPDI values showed: (i) ovariole number (0.09 and 0.04 in *D. buzzatii* and *D. koepferae*, respectively) was more plastic than body size (0.02 and 0.01 in *D. buzzatii* and *D. koepferae*, respectively) in both species; (ii) plasticity of ovariole number in *D. buzzatii* (0.09) was twice as much as in *D. koepferae* (0.04) (F_1,79_ = 5.89, *p* = 0.017) and iii) plasticity scores for body size were very similar across species (F_1,79_ = 0.66, *p* = 0.417).

### 3.2. Effect of Standard Diet (with No Cactus Additive) on Ovariole Number

In *D. buzzatii*, the mean number of ovarioles in females reared on a *O. sulphurea* semi-natural diet was 13% higher than in flies fed on a standard diet (Bonferroni Post Hoc test, *p* = 0.029), whereas no differences were found between females reared in *T. terscheckii* and on the standard diet. In *D. koepferae*, females raised on a standard diet had a substantially lower (33%) number of ovarioles than flies reared in *O. sulphurea* (Bonferroni Post Hoc test, *p* < 0.001) and in *T. terscheckii* (30%) (Bonferroni Post Hoc test, *p* < 0.001). Overall, after several generations under lab rearing, both species still responded to the addition of cactus albeit in different proportions.

### 3.3. Effect of Natural Substrates on Ovariole Number

A total of 85 and 109 females emerged from *O. sulphurea* and *T. terscheckii* substrates, respectively. All the females that emerged from *O. sulphurea* were *D. buzzatii*, whereas *D. koepferae* and *D. buzzatii* emerged from *T. terscheckii* in a 6:4 ratio. The mean ovariole number of *D. buzzatii* and *D. koepferae* emerged from natural substrates is shown in Table 4. In all the cases, the females that emerged from natural hosts had similar ovariole number than those reared on semi-natural media. Unfortunately, we could not estimate ovariole number in *D. koepferae* females emerging from the species’ secondary host, since flies of this species was not recovered from *O. sulphurea*.

## 4. Discussion

In this study, we sought to investigate the developmental plasticity in the number of ovarioles induced by a dietary effect of the natural host plants in cactophilic *D. buzzatii* and *D. koepferae*. Consistent with our hypothesis, the number of ovarioles was significantly higher in *D. buzzatii* females reared in *O. sulphurea*, the species’ main host [18,33]. In contrast, we found no differences in ovariole number between flies reared in different host plants in *D. koepferae*, even though this species showed a contrasting oviposition behavior across resources. In effect, the preference-performance hypothesis [30,31,32] seems to be valid only in *D. buzzatii* whereas, in *D. koepferae*, the preference for its primary natural host may respond to other factors than offspring performance. No association between preference and performance has been found in several insect-plant systems [39,40,41,42] and, in this particular case, given that this species is very sensitive to inter-specific competition with *D. buzzatii*, the preference for *T. terscheckii* might be explained as a strategy to avoid competition [43].

Our results showed that differences in ovariole number across rearing cacti are independent to variations in wing length (used as a measure of body size). Interestingly, a reduction in ovariole number has been found in females of *D. melanogaster* reared under juvenile diet restriction [7,9], suggesting that nutritional deficiencies might also account for the low number of ovarioles in *D. buzzatii* reared in the columnar cactus. Two recent studies reported the nutritional profiles of *O. sulphurea* and *T. terscheckii* [14,15]. The comparison between nutritional profiles shows quantitative differences in free sugars and fatty acids between cacti, in particular, the columnar cactus has lower amounts of oleic acid and disaccharides than *O. sulphurea*. Our results are in line with reports in other cactophilic *Drosophila* species, like *D. mojavensis,* in which subtle changes in sugar diets can dramatically affect developmental traits [44]. Since we used common baker´s yeast as a protein source in both media, a variation in protein content is unlikely to be the reason for the plastic responses. In addition, we confirmed that females emerging from natural substrates had similar ovariole number than females raised in semi-natural diet. Overall, our results indicate that rearing in alternative host plants affected *D. buzzatii* phenotypic plasticity, regardless the effect of specific cactophilic yeasts that flies feed on in nature.

It is of notice that, *T. terscheckii* contains toxic phenylethyaminic alkaloids as secondary compounds [19,20], thus we cannot rule out the possibility that toxic differences between cacti influences the development of reproductive structures. In this similar vein, the reduced number of ovarioles in *D. sechellia* is likely to be related to the high concentrations of octanoic acid in the specific host plant (*Morinda citrifolia*) that this fly exploits in nature [4]. Future studies will attempt to discern whether nutritional or toxicological rearing conditions (or both) are responsible for the plasticity in the reproductive capacity of *D. buzzatii* females.

Over the past years, the role of developmental plasticity in adaptive evolution has been receiving increasing attention [45,46,47]. In this vein, it has been recently suggested that nutritional plasticity of ovariole number has an adaptive value [10]. Only generalist *Drosophila* species, such as *D. melanogaster* and *D. simulans*, changed the number of ovarioles in response to diet variation. In contrast, specialist species, such as *D. sechellia* and *D. erecta*, exhibit similar ovariole number across different diets. Our results are in line with this pattern, since, on the one hand, the widespread species that has a wider niche (*D. buzzatii*) is highly plastic and, on the other, the endemic species that has a narrower niche (*D. koepferae*) is canalized, at least in these two host plants. The case of *D. koepferae* is certainly puzzling and deserves future attention, since flies do not achieve the highest ovariole number in the cactus from which it most frequently emerges in nature [21]. Thus, we advance the hypothesis that adaptive nutritional plasticity is the consequence of a trade-off between the number of ovarioles and egg size, as it was reported in artificially selected populations of *D. melanogaster* [48] and in other *Drosophila* species [49,50]. Species that, in response to poor diets, have the capacity to produce larger eggs [51] by reducing the number of ovarioles, can succeed in exploiting novel nutritional environments. Future efforts will be aimed at understanding the interplay between ovariole number plasticity, egg size and nutrition in cactophilic *Drosophila* species.

## 5. Conclusions

Though recently diverged (≅4.6 Mya) [11], *D. buzzatii* and *D. koepferae* have contrasting oviposition preferences between prickly pears and columnar cacti. In contrast, they do not exhibit particularly different ovariole numbers, although *D. buzzatii* expresses a much greater genetic variation for phenotypic plasticity in this reproductive trait. Such heritable variation may be the raw material for the evolution of adaptive plasticity in heterogeneous environments, as those represented by the diverse natural hosts.

## Figures and Tables

**Table 1:** Analysis of variance testing for differences in ovariole number for both species.

Source	d.f.	MS	F	P
Species	1	0.001	0.001	0.995
Cactus	1	0.021	2.859	0.140
Species × Cactus	1	0.001	0.171	0.693
Line (S)	6	0.022	2.996	0.101
Line (S) × Cactus	6	0.007	4.235	**0.005**
Wing Length	1	0.002	1.596	0.209
Error	117	0.001		

**Table 2:** Analysis of variance testing for differences in ovariole number in *D. buzzatii*.

Source	d.f.	MS	F	P
Line	3	0.048	3.723	0.150
Cactus	1	0.014	1.273	0.336
Line × Cactus	3	0.014	7.539	**0.001**
Wing Length	1	0.001	0.806	0.372
Error	79	0.002		

**Table 3:** Analysis of variance testing for differences in ovariole number in *D. koepferae.*

Source	d.f.	MS	F	P
Line	3	0.006	2.119	0.269
Cactus	1	0.004	1.145	0.365
Line × Cactus	3	0.003	2.623	0.067
Wing Length	1	0.001	1.048	0.309
Error	71	0.001		

**Table 4:** Mean ovariole number (X) and standard deviation (SD) in females emerging from natural hosts and reared on semi-natural media.

Species-by-Cactus	Natural Host (X ± SD)	Semi-Natural (X ± SD)	*F*	*P*
*D. buzzatii* in *O. sulphurea*	41.1 ± 2.50	43.4 ± 5.48	1.954	0.167
*D. buzzatii* in *T. terscheckii*	38.9 ± 6.38	40.4 ± 7.50	0.391	0.534
*D. koepferae* in *T. terscheckii*	43.1 ± 3.26	42.5 ± 3.27	0.255	0.615
